# Effective Protein–Ligand Docking Strategy via Fragment Reuse and a Proof-of-Concept Implementation

**DOI:** 10.1021/acsomega.2c03470

**Published:** 2022-08-19

**Authors:** Keisuke Yanagisawa, Rikuto Kubota, Yasushi Yoshikawa, Masahito Ohue, Yutaka Akiyama

**Affiliations:** ‡ Department of Computer Science, School of Computing, Tokyo Institute of Technology, Meguro-ku, Tokyo 152-8550, Japan; § AIST-Tokyo Tech Real World Big-Data Computation Open Innovation Laboratory (RWBC-OIL), National Institute of Advanced Industrial Science and Technology, Tsukuba, Ibaraki 305-8560, Japan

## Abstract

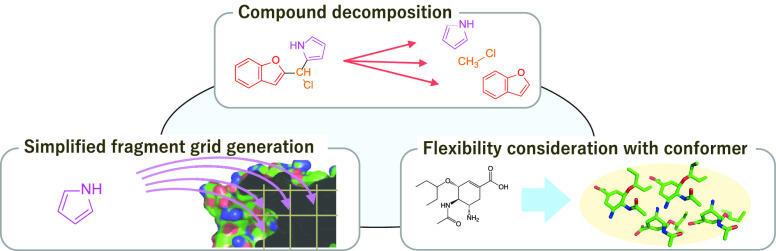

Virtual screening is a commonly used process to search for feasible drug candidates from a huge number of compounds during the early stages of drug design. As the compound database continues to expand to billions of entries or more, there remains an urgent need to accelerate the process of docking calculations. Reuse of calculation results is a possible way to accelerate the process. In this study, we first propose yet another virtual screening-oriented docking strategy by combining three factors, namely, compound decomposition, simplified fragment grid storing *k*-best scores, and flexibility consideration with pregenerated conformers. Candidate compounds contain many common fragments (chemical substructures). Thus, the calculation results of these common fragments can be reused among them. As a proof-of-concept of the aforementioned strategies, we also conducted the development of REstretto, a tool that implements the three factors to enable the reuse of calculation results. We demonstrated that the speed and accuracy of REstretto were comparable to those of AutoDock Vina, a well-known free docking tool. The implementation of REstretto has much room for further performance improvement, and therefore, the results show the feasibility of the strategy. The code is available under an MIT license at https://github.com/akiyamalab/restretto.

## Introduction

During the early stages of drug discovery research, drug candidates are explored and screened from a compound database.^1^ Compound databases have expanded rapidly in recent years; for instance, approximately 1.4 billion compounds have been registered in the ZINC20 database,^2^ a database of purchasable compounds. Structure-based virtual screening (SBVS) is widely used in the early stages of drug discovery to perform an efficient screening of a huge number of compounds.^3^ However, SBVS is extremely computationally challenging to apply to very large chemical databases, such as ZINC20. The speed of the rigid-protein, flexible-ligand docking process is not sufficiently fast (1–500 s per compound^4−6^) to evaluate more than a billion compounds in a compound library. Thus, further acceleration is warranted.

Most docking tools are used to evaluate a pair of a target protein and a compound independently from other candidate compounds; all intermediate results are discarded before conducting the evaluation of the next compound. Virtual screening relies on a substantial number of dockings performed for a target protein, and thus, there is an urgent need to accelerate the process of docking calculations. The most intuitive way to accelerate the process is via the parallelization of many evaluations, which are completely independent. However, discarding the intermediate results is not efficient, and it is possible to further accelerate the process by focusing on the reuse of intermediate results, rather than discarding them. Indeed, compound libraries contain a substantial number of derivative compounds that demonstrate common chemical substructures (hereafter referred to as fragments). Previously we decomposed 28 629 602 compounds in the ZINC12 database and found only 263 319 unique fragments.^7^ Furthermore, several functional groups and moieties are common among compounds. These tendencies are consistent with combinatorial chemistry used in the compound generation process. The advantage of using the commonality between compounds to accelerate calculations is based on the following principle: the larger the number of compounds, the higher the degree of commonality because of the saturation of the appearance of unique fragments. This approach is particularly suitable for performing virtual screening with billions of compounds.

Spresso^7^ is an exemplary method based on the commonality of fragments. The fundamental idea behind Spresso is that binding affinities of a few important fragments are key elements in the binding of a whole compound. The Spresso procedure comprises three steps. First, the compounds are decomposed into fragments. Each of the fragments is then docked to the target protein individually. Finally, compounds are evaluated on the basis of the docking scores of the fragments. Spresso has been shown to accelerate calculations approximately by 200-fold; however, it only calculates compound scores and cannot output binding complex structures of proteins and compounds.

Conversely, there have been several fragment-based docking tools available, such as FlexX,^8^ DOCK 6,^9^ and eHiTS,^10^ which can be used to output binding complex structures. All the existing fragment-based docking tools employ incremental construction strategy.^11^ For instance, FlexX employs incremental building-up strategy. The base fragment is first docked into the active site, then the remaining fragments are incrementally built up with consideration of ligand flexibility. eHiTS utilizes a maximum clique search to reconstruct a compound’s structure from its fragments’ poses. Particularly, eHiTS has already implemented the reuse of fragment docking results using the SQL database, and has demonstrated the achievement of 2- to 4-fold accelerations. However, maximum clique search is an NP-hard problem, and the application of eHiTS requires several minutes of calculations per compound. A series of tools, namely, DAIM, SEED, and FFLD, reduces the calculation cost by selecting three important fragments (triplet) from a compound to effectively search docking poses.^12^ However, the series of tools employs a genetic algorithm to generate conformations of a compound, resulting in a considerably high calculation cost. Therefore, we must consider more effective ways to construct a compound’s pose from the fragment docking results.

One of the ways to accelerate the process is via conformer docking, which considers the flexibility of a compound by using pregenerated conformers of the compound. Each conformer reflects the prior knowledge of the compound tertiary structure including bond length, bond angles, and torsion angles. Furthermore, several conformer generators such as OMEGA^13^ enumerate conformers with consideration of torsion angle statistics of the binding states. Therefore, conformer docking tools do not need to consider compound flexibility by themselves, resulting in substantial acceleration. FRED^14^ is a conformer docking tool that employs fast shape-matching of a pregenerated conformer and a protein surface. However, it conducts compound-based docking and does not use fragment commonality. Therefore, the combination of conformer generation that considers possible conformation space attached to the original compound and fragment-based docking that utilizes the fragment commonality, may enhance the speed of docking, especially for a large chemical database.

In this paper, we propose another virtual screening-oriented, fragment-based docking strategy for evaluating a huge number of compounds against a single target protein. This strategy focuses on the fact that compounds contain many common fragments. The data on the results of the calculations for these fragments can be stored and reused, along with flexibility considerations, through conformer generation. To the best of our knowledge, this is the first study to discuss suitable strategies that consider chemical substructure commonality during the protein–ligand docking process though each factor is inspired by existing methods.

We implemented a proof-of-concept of the virtual screening-oriented, fragment-based docking strategy, named REstretto (REuse of sub-STRuctures as an Effective Technique for protein–ligand docking TOol). REstretto is operated with the AutoDock Vina scoring function. However, it uses a different search algorithm from that of AutoDock Vina. REstretto searches the best poses through a comprehensive, systematic search using pregenerated conformers, while AutoDock Vina searches them through an iterated local search of the rotation of the chemical bonds. As conformers are used, REstretto does not need to consider compound flexibility unlike FlexX or eHiTS, which employ incremental construction algorithm. Our findings demonstrated that fragment-based REstretto achieved a comparable performance as that of compound-based AutoDock Vina in terms of execution time and accuracy with tens of thousands of compounds. We concluded that the proposed strategy is promising because further acceleration is guaranteed with a substantial increase in the number of compounds.

## Materials and Methods

### Fundamental Idea for Accelerating Virtual Screening-Oriented Docking

**Figure 1:**
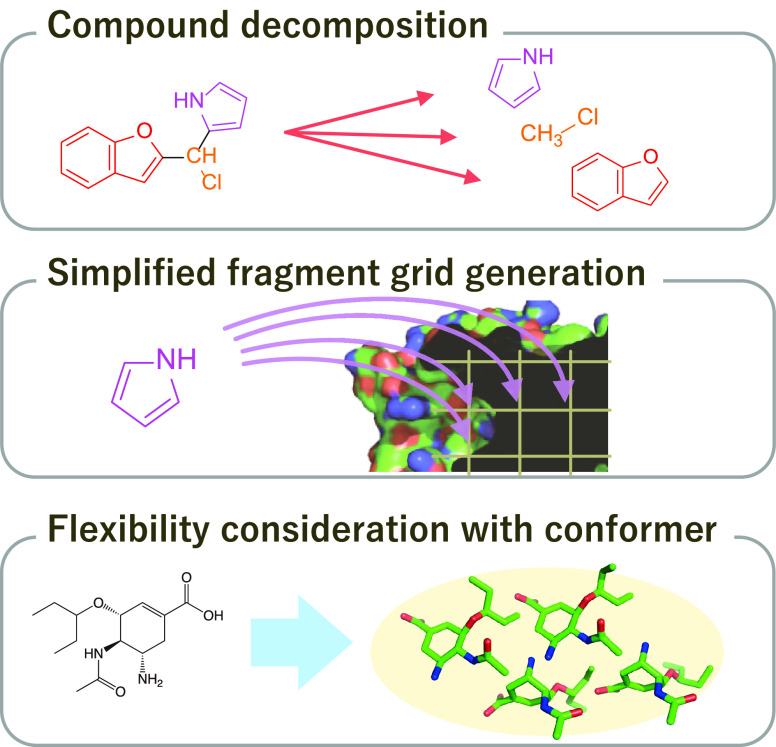
Three factors of the fundamental ideas.

As mentioned in the previous section, our fundamental idea for acceleration is to enable the reuse of common intermediate calculation results among huge number of docking processes. Thus, it is important to consider ways to define the intermediate results and the manner in which we can use them efficiently. In this article, we propose a fragment-based docking procedure with three factors, namely compound decomposition, simplified fragment grid generation, and flexibility consideration with conformers. The three factors are illustrated in Figure 1.

#### Compound Decomposition

The most important factors of fragment-based docking comprise the reusability and effectiveness of the intermediate results. For instance, the atom grid or the docking scores of an atom among binding pockets, is the most reusable and widely used.^15,16^ However, the time required for compound evaluations performed with atom grids is proportional to the number of atoms in the compound. The time required for compound evaluations performed using intermediate results of fragments is proportional to the number of fragments in the compound under certain conditions, including that a fragment must not be present with internal degrees of freedom (see Text S1 in the Supporting Information). The calculation cost of an intermediate result will exponentially increase with the degrees of freedom of a fragment because of its flexibility. Therefore, the best feature of a fragment in terms of an intermediate result is the absence of internal degrees of freedom and the presence of many atoms.

#### Simplified Fragment Grids Storing *k*-Best Fragment Rotations

The next factor is the representation of the intermediate results. Many poses between a target protein and a compound are evaluated to search for the best poses. Thus, an intermediate result needs to include the docking scores of each position. We extend an atom grid, which is an intermediate result of an atom, to a fragment grid, which is an intermediate result of a fragment. The use of fragment grids reduces the calculation cost.

However, the memory consumption of a fragment grid is a serious challenge that needs to be overcome; the docking score of a fragment depends not only on the position in the binding pocket, but also on its rotation, while an atom is rotation-invariant. Rotation must be considered to generate a fragment grid. The number of types of fragments is >200 000 at most, resulting in tens of terabytes of memory consumption with 60 rotation patterns for each fragment (as introduced in eHiTS^17^). Thus, storing all fragment grids with all rotations to the capacity-limited random access memory (RAM) is quasi-impossible. An option to overcome this challenge is storing only a part of the best scores among the patterns of rotations at each position; we call this a simplified fragment grid. The *k*-best storing strategy considerably reduces memory consumption and enables the storage of all fragment grids in the memory. To calculate a compound score, information on the score of the specific rotation most similar to the rotation to be evaluated is retrieved. Note that the prediction accuracy may decrease by this simplification, and thus, we compare the accuracy of the method following the use of simplified and full (storing-all) fragment grids in the Discussion.

#### Flexibility Consideration with Pregenerated Conformers

The last factor of fragment-based docking is the reconstruction of a compound while considering its flexibility. The valid relative positions of fragments are judged by considering the acceptable conformational space attached to the original compound. At times, this validity relationship is expressed using a graph structure in the incremental construction strategy. FlexX,^8^ for instance, reconstructs a compound’s structure using a tree search algorithm. Furthermore, eHiTS^10^ reconstructs a compound’s structure by solving the maximum clique problem, which is known as an NP-hard problem. These two algorithms are used to tackle a difficult task without using prior information on the compound’s conformations. Indeed, all rotatable bonds have an energetic preference for dihedral angles. This suggests that the prior knowledge of feasible compound conformation may make the task easier. Unfortunately, to date, all implementations of incremental construction have not or have hardly considered the prior knowledge. A good way to take into consideration the prior knowledge of the compound conformation is conformer generation. The generation of a conformer effectively limits the search space such as through the exclusion of inappropriate torsional rotation, and internal collision of atoms. Here, the implementation of FRED^14^ involves the conformer docking strategy and considers the input conformers as rigid structures. The positions and rotations of each conformer are the only aspects that need to be considered with this strategy, thus resulting in a significant acceleration of the docking phase.

### Overview of a Proof-of-Concept, REstretto

**Figure 2:**
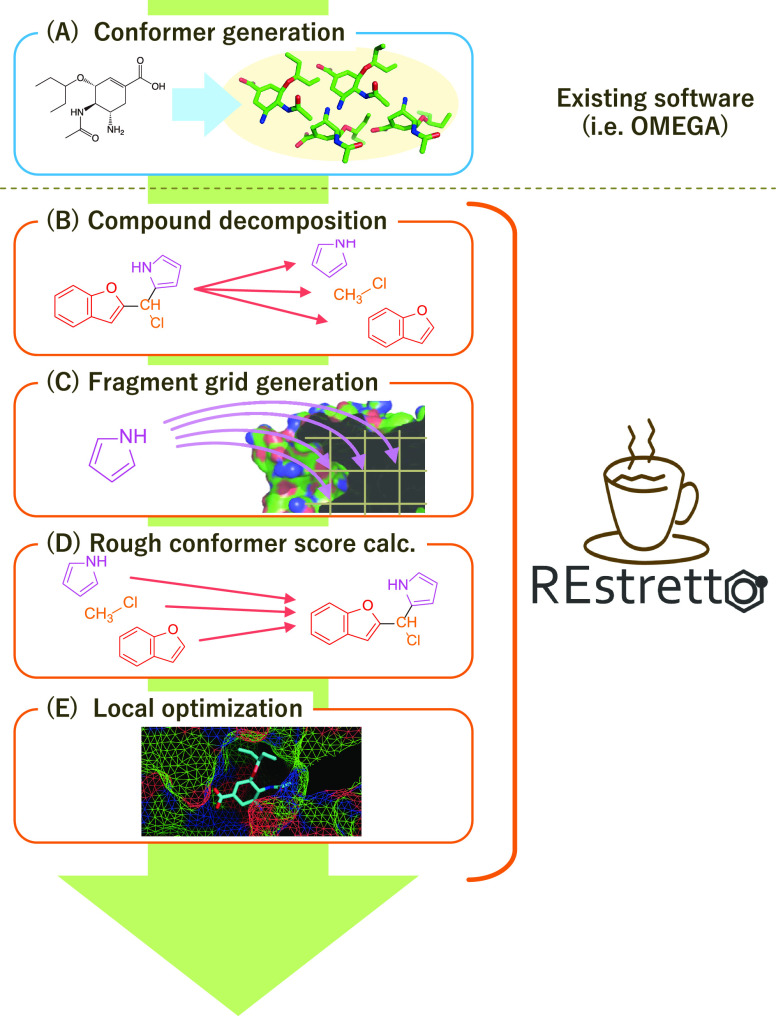
Workflow of the REstretto, a proof-of-concept implementation of the proposed strategy.

The feasibility and advantages of the proposed virtual screening-oriented, fragment-based docking were explored above. However, a quantitative evaluation of its effectiveness is necessary. We thus implemented REstretto as a proof-of-concept software of the fundamental idea. The workflow of REstretto is shown in Figure 2. REstretto was implemented using C++ with OpenBabel^18^ and Boost libraries. REstretto employs the scoring function of AutoDock Vina.

REstretto consisted of the following procedure and was based on the use of one central processing unit (CPU) core for all processing experiments.

1. Hundreds of conformers of an input compound are generated.
2. The compound is partitioned into fragments.
3. All unique fragments found in the compound are enumerated.
4. Dedicated fragment grids against the target protein are generated. Fragment grids are reused if they have already been generated during a previous evaluation of another compound.
5. Each conformer of the compound is evaluated using information regarding the relative position of its fragments. Rough conformer scores for all possible placements on a predetermined docking region are exhaustively calculated.
6. Feasible conformer placements with high rough conformer scores are selected, and local optimization of the protein-conformer complex structures is performed via a detailed evaluation.
7. A specified number of the best complex structures of the compound are output as the result.

The inputs of REstretto are conformers of compounds; thus, it is necessary to enumerate the conformer using a conformer generator. Each step illustrated in Figure 2 is described in the following sections.

#### Conformer Generation (Figure 2A)

In our strategy, we reconstructed compound structures based on pregenerated conformers rather than incremental construction. Many conformer generators have been proposed, such as ConfGen/ConfGenX,^19^ iCon (implemented in LigandScout^20^), and OMEGA.^13^ In this study, we used OMEGA (version 2.5.1.4, OpenEye Scientific Software, Inc.) because it is one of the most powerful conformer generators.^21^ We generated up to 200 conformers per compound, which is the default parameter of OMEGA.

#### Compound Decomposition (Figure 2B)

The fragments should not have an internal degree of freedom, as previously mentioned; hence, each compound was decomposed into fragments using the same algorithm as Spresso.^7^ The decomposition algorithm is briefly described below:

1. Each atom in a compound is defined as a fragment at the initial state.
2. Each bond in a compound is labeled according to whether it is rotatable.
3. Adjacent atoms with a nonrotatable bond are united in a fragment.
4. Atoms constituting a ring are united in a fragment.
5. For each fragment consisting of two or more atoms, adjacent solitary fragments (fragments consisting of an atom) are united if the solitary fragments contain up to one additional adjacent fragment.

Fragments having the same canonical SMILES were treated as the same fragment. Canonical SMILES can distinguish stereoisomers. Further information about the decomposition can be found in the original literature.^7^

#### Fragment Grid Generation (Figure 2C)

The fragment grid, or the fragment docking scores around the pocket of a protein, were then calculated at equal intervals (0.25 Å increments as a default) in three-dimensional space. We used a dodecahedron-based fragment rotation, initially introduced in eHiTS,^10^ which has 60 rotation patterns. As described above, the AutoDock Vina energy score function was used.

**Figure 3:**
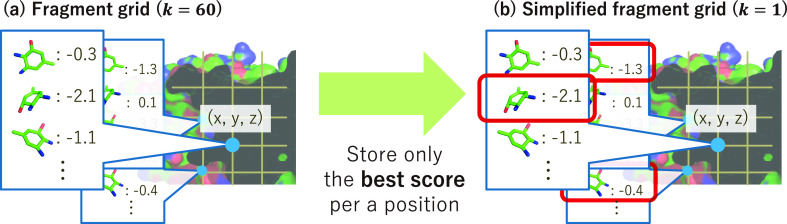
Construction of the simplified fragment grid. (a) Fragment grid containing scores of all rotations. (b) Simplified fragment grid containing the 1-best (*k* = 1) score among rotations per position.

A fragment grid would occupy approximately 400 MB of memory for a cubic grid containing 121 points on a side (30 Å per side, spaced at 0.25 Å) with 60 rotation patterns being stored. Thus, in this study, we used a simplified fragment grid with a variable *k* = 1, storing only the best score among the rotations (Figure 3), along with optimization of the simplified fragment grid packing into capacity-limited memory space.^22^ Despite the significant loss of information from the original grid conserving all rotations (*k* = 60), the rough conformer score calculated with the simplified fragment grid was no worse than the genuine score (the rough score is considered as a lower bound, where lower values are better). The nature of the rough score is appropriate for selecting the binding poses for subsequent local optimization, since the final aim was to obtain a few best poses per compound. The size of the simplified fragment grid (*k* = 1) was only <7 MB per fragment.

#### Calculation of the Rough Conformer Score from Simplified Fragment Grids (Figure 2D)

**Figure 4:**
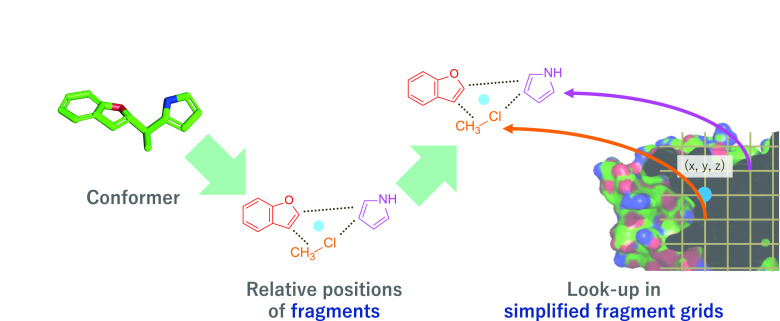
Rough conformer score calculation.

Each conformer was roughly scored using a simplified fragment grid. The scoring flow is shown in Figure 4. First, the relative position of each fragment from the center of gravity was determined for a conformer. Second, the score of each fragment was looked-up in its simplified fragment grid, followed by the rough conformer score calculation (which involved summation of the simplified fragment grid scores). The rough conformer scores described above were exhaustively calculated using translation and rotation. Information on the specific number (2000 by default) of the best binding poses among all conformers of a compound was stored. It should be noted that the conformer was treated as a rigid structure in REstretto because the conformation space was sufficiently covered by pregenerated conformers in most cases; consequently, incremental construction for flexible ligand docking was not performed.

#### Local Optimization of the Best Initial Poses (Figure 2E)

For all poses selected in the previous step, local optimization of the conformer placement including translation and rotation was performed using atom grids to obtain more rigorous scores. This step was performed using the hill-climbing method. First, several poses (200 by default) that were slightly and randomly translated or rotated from a current pose were generated, and their docking scores were calculated. The internal degree of freedom was not considered in current implementation. Second, the current pose was updated with the best pose obtained among them. These two steps were repeated until no improvement in the scores was achieved. After the local optimization of all poses of a compound, the best poses and their scores were output.

Local optimization process is common among not only fragment-based docking but also compound-based docking. Thus, the efficiency of the local optimization is beyond that of the proof-of-concept, and we utilized the simple hill-climbing method, which is less efficient than gradient-based optimization.

### Data Set

To benchmark the performance of REstretto, we utilized the diverse subset of the Directory of Useful Decoys, Enhanced (DUD-E diverse subset)^23^ as a data set. The DUD-E diverse subset consists of eight targets including kinase, G-protein coupled receptor, and protease, and is provided as a subset representing all 102 targets in DUD-E. Because of its diversity, it was also used for evaluating docking scoring functions.^24^ Each target consists of a cocrystal structure, active compounds, and decoy compounds.

First, all compounds in each target were preprocessed using the LigPrep module of the Schrödinger suite (version 2018–1). LigPrep is a commercial tool used to generate up to 32 compound states including stereoisomeric and ionization states at pH 7.0 ± 2.0, with default parameters. The generated states were independently used for comparative purposes. Note that on average, approximately two states were generated from a compound in this experiment.

### Performance Comparison with Other Methods

To prove the effectiveness of our strategy, we compared the results with AutoDock Vina, because REstretto uses the same scoring function, allowing us to analyze the potential differences between the two methods. AutoDock Vina is one of the most accurate free docking simulation tools.^25^ The parameters related to the search space and exhaustiveness are described in Experimental Setups. Additionally, we have indicated the results of Glide (on Schrödinger suite version 2018–3, Schrödinger, LLC),^4^ which is a proprietary docking simulation software package, for reference. We could not implement the score function of Glide because it is undisclosed; the accuracy and even the calculation speed are markedly affected by the differences in the scoring functions.

### Metrics

We evaluated their accuracy with respect to two metrics, namely area under the receiver operating characteristic curve (ROC-AUC) and enrichment factor (EF). We also evaluated their execution time.

The ROC curve was plotted according to the true positive rate and false positive rate, with various docking score thresholds, depicted on the horizontal and vertical axes, respectively. The ROC-AUC was greater than 0.5 when the ROC curve was above the diagonal line, with random predictions leading to the achievement of an ROC-AUC value of 0.5.

EF can be calculated using the below-mentioned formula:
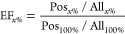
where Pos_*x*%_, All_*x*%_, Pos_100%_, and All_100%_ represent the number of active compounds in the top *x*% of screened compounds, the number of compounds in the top *x*% of screened compounds, the total number of active compounds, and the total number of compounds, respectively. Note that EF_*x*%_ = 1.0 means a random prediction. We calculated EF_1%_ and EF_10%_ in this study.

The execution time was output using REstretto and Glide, whereas it could not be output using AutoDock Vina. We utilized the execution time information, when available; otherwise, we utilized the Linux date command. All docking tools were used with a single CPU core per thread, unless otherwise stated.

### Experimental Setups

A docking region box must be explicitly specified for both REstretto and AutoDock Vina. The center coordinates and, the lengths on each side of the docking region cubes were determined using eBoxSize^26^ based on the position and size of the cocrystallized ligand in the complex structure of the target protein. Table 1 shows the center coordinates and the lengths on each side of docking region cubes. Note that the box was not specified for Glide because it can determine its own box from a complex structure by default.

**Table 1:** Protein Data Bank (PDB) ID and Box Information Determined Using the eBoxSize^26^ of Each Target

target	PDB ID	box center	docking region
AKT1	3CQW	(5.99 Å, 3.01 Å, 17.34 Å)	14 × 14 × 14 Å^3^
AMPC	1L2S	(80.84 Å, 5.01 Å, 31.29 Å)	18 × 18 × 18 Å^3^
CP3A4	3NXU	(36.69 Å, −15.69 Å, 29.69 Å)	24 × 24 × 24 Å^3^
CXCR4	3ODU	(20.17 Å, −7.83 Å, 70.62 Å)	18 × 18 × 18 Å^3^
GCR	3BQD	(39.85 Å, 30.29 Å, 9.33 Å)	22 × 22 × 22 Å^3^
HIVPR	1XL2	(20.14 Å, −2.72 Å, 18.30 Å)	20 × 20 × 20 Å^3^
HIVRT	3LAN	(9.53 Å, 12.41 Å, 17.43 Å)	18 × 18 × 18 Å^3^
KIF11	3CJO	(17.81 Å, 16.12 Å, 109.27 Å)	20 × 20 × 20 Å^3^

All tools contain parameters and modes to enable adjustments for the granularity of the search. The exhaustiveness parameter of AutoDock Vina is a typical example, and we tested exhaustiveness = 1 (ex = 1) and exhaustiveness = 8 (ex = 8). For Glide, we evaluated the high throughput virtual screening mode (Glide HTVS) and standard precision mode (Glide SP). The other parameters of Glide were their default values. Finally, we tested REstretto using its default parameters.

In addition to the above-mentioned settings, it was necessary to limit the number of stereoisomeric and ionization states of the compounds in an execution because of the limited execution time per run of the computer environment. If the total number of compound states in an original compound set exceeded 10 000, the said set was split into a number of sets containing up to 10 000 compound states each after subjection to sorting; docking simulations were then performed independently. REstretto differs from the existing tools, such as AutoDock Vina and Glide, in that it reuses simplified fragment grids within a group of compounds. Therefore, this splitting process reduces the speed of REstretto.

### Computing Environment

All calculations were conducted using the TSUBAME3.0 supercomputing system at Tokyo Institute of Technology, Japan. Each node comprises two Intel Xeon E5–2680 V4 CPUs (14 cores per CPU) and 256 GB of RAM.

## Results

### Compound Decomposition

The number of the fragment types of actives and decoys for each target in the DUD-E diverse subset was counted as the result of the compound decomposition. The total number of fragments, the number of types of fragments, and the number of compounds in each target are shown in Table 2. The number of fragment types of each target of DUD-E diverse subset was less than the number of compounds, indicating that there were many common fragments. The ratio of the number of fragment types to the number of compounds tended to decrease with an increase in the number of compounds.

**Table 2:** Number of Compounds, Fragments, And Types of Fragments for Each Target in the DUD-E Diverse Subset^a^

target	no. of compds	no. of fragments	no. of types of fragments
AKT1	16 743	97 119 (×5.8)	8536 (×0.51)
AMPC	2898	11 848 (×4.1)	2843 (×0.98)
CP3A4	11 970	69 612 (×5.8)	8348 (×0.70)
CXCR4	3446	18 801 (×5.5)	2848 (×0.83)
GCR	15 258	73 662 (×4.8)	8378 (×0.55)
HIVPR	36 286	247 065 (×6.8)	11 098 (×0.31)
HIVRT	19 229	88 673 (×4.6)	11 129 (×0.58)
KIF11	6966	33 601 (×4.8)	5415 (×0.78)

a The ratios to the number of compounds are shown in parentheses.

We also analyzed the statistical data of all compounds among the DUD-E diverse subset targets for further investigation of the relationship between the increase in the number of compounds and the increase in the number of fragment types. The number of compounds, fragments, and types of fragments were 112 796,  640 381 (×5.7), and 30 535 (×0.27), respectively (the ratio to the number of compounds are shown in parentheses). This trend was also supported by the results.

### Accuracy of the Docking Simulation

Docking simulations using REstretto, AutoDock Vina (with two different exhaustiveness parameters), Glide HTVS, and Glide SP were performed using the DUD-E diverse subset, and the prediction accuracy of each method was calculated. The average values of ROC-AUC, EF_1%_, and EF_10%_ over the targets are listed in Table 3. More specifically, the metrics for each target and all ROC curves are shown in Tables S2 and S3 and Figures S1 and S8. The average ROC-AUC of the proposed method was 0.657, which was slightly higher than 0.644, i.e., the value obtained with AutoDock Vina (ex = 8). The EFs of REstretto tended to be lower than those of AutoDock Vina. It should also be noted that the accuracy of Glide SP mode was better than that of AutoDock Vina and REstretto. The combinational use of the “softer” scoring function forgiving imperfect poses and the hierarchical filtering of the docking poses to omit inappropriate poses effectively contribute to the better performance of Glide SP.^4^

**Table 3:** Average Values of ROC-AUC, EF_1%_, and EF_10%_ over the DUD-E Diverse Subset^a^

		AutoDock Vina		Glide	
metrics	REstretto	ex = 1	ex = 8	HTVS	SP
ROC-AUC	**0.657**	0.638	0.644	0.667	0.725
EF_1%_	5.4	7.2	**7.8**	8.6	16.5
EF_10%_	2.6	**2.7**	**2.7**	3.1	4.3

a The best values between REstretto and AutoDock Vina are shown in bold.

### Execution Time of the Docking

The average execution times per compound with active and decoy compounds in the DUD-E diverse subset are shown in Table 4. REstretto demonstrated an execution time comparable to that of AutoDock Vina. REstretto was faster for serine/threonine-protein kinase AKT (AKT1), which demonstrated a smaller ratio (×0.51) of the number of types of fragments to the number of compounds. In contrast, REstretto was slower for AmpC β-lactamase (AMPC), which demonstrated a larger ratio (×0.98) of the number of types of fragments to the number of compounds.

**Table 4:** Execution Times Per Compound with Active and Decoy Compounds in the DUD-E Diverse Subset (CPU core s)^a^

	execution time (CPU core s)				
		AutoDock Vina		Glide	
DUD-E target	REstretto	ex = 1	ex = 8	HTVS	SP
AKT1	**22.95**	48.12	619.87	0.46	12.04
AMPC	23.86	**14.02**	168.39	0.57	5.93
CP3A4	41.05	**25.52**	304.86	1.23	21.83
CXCR4	**33.02**	33.48	404.03	1.82	32.91
GCR	28.83	**17.67**	236.56	0.39	8.69
HIVPR	31.84	**30.83**	376.09	0.68	17.47
HIVRT	19.96	**13.66**	180.75	0.27	5.51
KIF11	34.08	**23.20**	276.24	0.66	13.63
average	29.04	**27.30**	341.89	0.63	13.84

a The fastest time between REstretto and AutoDock Vina for each DUD-E target is shown in bold. Note that AutoDock Vina (ex = 8) was calculated with 12 cores and the execution times of all cores were accumulated.

## Discussion

### Comparison of the Accuracies of the Full Fragment Grid and Simplified Fragment Grid

Our simplified fragment grid significantly reduced memory consumption; however, the degradation of accuracy must be discussed. We compared the accuracy of full fragment grids (*k* = 60) and simplified fragment grids (*k* = 1). Interestingly, the accuracy (ROC-AUC) did not degrade with the use of the simplified fragment grid with five DUD-E targets (Table S5). This result suggests that simplified fragment grids cause only a subtle change in the initial poses of the conformers in many cases. As these subtle changes may vanish through local optimization, the final outputs would not greatly differ from those obtained with the use of full fragment grids.

### Byproduct of the Proposed Strategy

As we described, the proposed strategy enables the reuse of intermediate results. Additionally, the strategy provides byproducts, namely, (1) fragment-based prescreening of compounds with fragment knowledge and (2) an orientation-aware scoring function.

The fragment-based prescreening will accelerate the whole docking procedure by filtering out nonfeasible conformers and compounds in the earlier step. For instances, Spresso,^7^ a fast prescreening tool, only considers individual docking scores of fragments and does not reconstruct the compound structure, resulting in the <10 ms evaluation per compound. FFLD,^27^ another fragment-based method, considers the relative positions of three fragments and selects acceptable conformers.

Meanwhile, the application of the orientation-aware scoring function may improve the screening accuracy though we utilized the orientation-independent AutoDock Vina scoring function. Existing physics-based or empirical energy score functions mostly depend on the atom grid. This limits out some important scoring function, for instance, the π–π interaction. More importantly, atom grid limits angle-aware hydrogen bonding. The position of the lone pairs of hydrogen bond acceptor atoms of a target protein can be determined, whereas the positions of the lone pairs of a compound cannot be determined since an atom grid does not provide any bonding information. The fragment grid solves this problem because the fragment provides bond information. Thus, the positions of lone pairs can be determined even for a compound.

### Breakdown of the Execution Time of REstretto

To determine the effectiveness of fragment-based docking and identify areas with room for improvement, we measured the breakdown of the execution time of REstretto with DUD-E compounds as shown in Table 5. A considerable portion of execution time was consumed in (C) fragment grid generation and (E) local optimization. Indeed, fragment grid generation comprised more than half of the execution time for all targets except AKT1. The execution time of (C) was proportional to the number of types of fragments. The ratio of AKT1 was the least among the eight targets, which is the reason for the high speed of REstretto for AKT1. Another reason why the execution time of (C) of AKT1 was smaller than that of the others was the size of the docking region. On the basis of the information presented in Table 1, it was inferred that the docking region of AKT1 was the smallest of the eight targets, with a cube of 14 Å on each side. This markedly affected the execution time of (C) because all possible positions inside the box were calculated and stored as a fragment grid.

**Table 5:** Breakdown of the Average Execution Time of Each Step of REstretto per Compound State (CPU core s)^a^

	average execution time (CPU core s)			
	(B) structure decomposition	(C) grid generation	(D) rough conformer scoring	(E) local optimization
target	*O*(|*T*_C_|)	*O*(∑_*f*∈*F*_|*A*_*f*_|)	*O*(|*T*_F_|)	*O*(|*T*_C_|)
AKT1	1.23 (11.4%)	3.26 (30.2%)	0.22 (2.0%)	5.36 (49.7%)
AMPC	N/A	N/A	N/A	N/A
CP3A4	1.31 (5.6%)	15.00 (63.7%)	0.77 (3.3%)	5.88 (25.0%)
CXCR4	N/A	N/A	N/A	N/A
GCR	0.93 (5.0%)	10.72 (57.8%)	0.48 (2.6%)	5.99 (32.3%)
HIVPR	1.73 (8.9%)	10.14 (52.3%)	0.50 (2.6%)	6.30 (32.5%)
HIVRT	0.55 (4.1%)	7.21 (53.7%)	0.22 (1.6%)	5.13 (38.2%)
KIF11	0.99 (5.6%)	10.01 (56.6%)	0.39 (2.2%)	5.82 (32.9%)

a This shows the calculation time for 10 000 compounds. The breakdown was not applicable (N/A) for the targets AMPC and CXCR4, as they comprised less than 10 000 compounds. |*T*_C_|, *F*, |*A*_*f*_|, and |*T*_F_| represent the total number of compounds, the set of the types of fragments, the number of atoms in a fragment *f*, and the total number of fragments among all compounds, respectively.

In the experiments, the ratios of the number of types of fragments to the number of compounds were less than 1.0 for all cases (×0.31 to ×0.98, Table 2). The ratios of the number of types of fragments to the number of compounds will be much less; a previous study revealed that >28 million compounds could be expressed using only <270 000 fragments. Therefore, the ratio can be decreased by less than 0.01, resulting in the achievement of >100-fold acceleration. This acceleration does not happen to AutoDock Vina, and thus it is a substantial advantage of REstretto.

If the number of compounds increases, (E) local optimization will emerge as the most time-consuming step in REstretto. Since local optimization was beyond the scope of the proof-of-concept, we implemented a simple, hill-climbing method. However, a more sophisticated, gradient-based optimization is warranted to highlight the real ability of the proposed strategy.

### Search Algorithm Comparison between REstretto and AutoDock Vina

REstretto used the same energy score function as AutoDock Vina; therefore, most differences in accuracy depended on the difference between the search space and the search algorithm. REstretto and AutoDock Vina differ with respect to three main factors in the search space and algorithm: (1) the way in which they consider the internal degrees of freedom of a compound, (2) the size of the physical search space, and (3) the search algorithm.

(1) **The way in which they consider internal degrees of freedom:** Whereas AutoDock Vina considers the internal degrees of freedom (rotation of single bonds), REstretto considers it via conformer generation. Therefore, from this perspective, AutoDock Vina can be used to perform a more detailed search than REstretto can.
(2) **The size of physical search space:** AutoDock Vina is used to search for coordinates and rotations such that all atoms of a compound are in the given search space. In contrast, we defined the search space of REstretto such that only the center of gravity of a compound was in the given search space, except for the existence of any atom at a distant location from the space (5 Å by default). Therefore, REstretto is used to search for a wider physical space than AutoDock Vina. The increase in execution time of REstretto, with increasing search space size, is less intense than that observed with AutoDock Vina, thereby leading to their differing search space definitions.
(3) **Search algorithm:** REstretto is based on comprehensive searches among spaces using simplified fragment grids, whereas AutoDock Vina is based on an iterated local search that begins from randomly generated poses. Therefore, AutoDock Vina focuses on a randomly selected local space, whereas REstretto first begins a search of the entire space at a certain granularity and moves into local optimization. These two algorithms have different advantages; the iterated local search by AutoDock Vina can find a local minimum efficiently, whereas the exhaustive search by REstretto can find several feasible docking poses.

### Wider Search Space of REstretto Enables Accurate Prediction for AKT1

**Figure 5:**
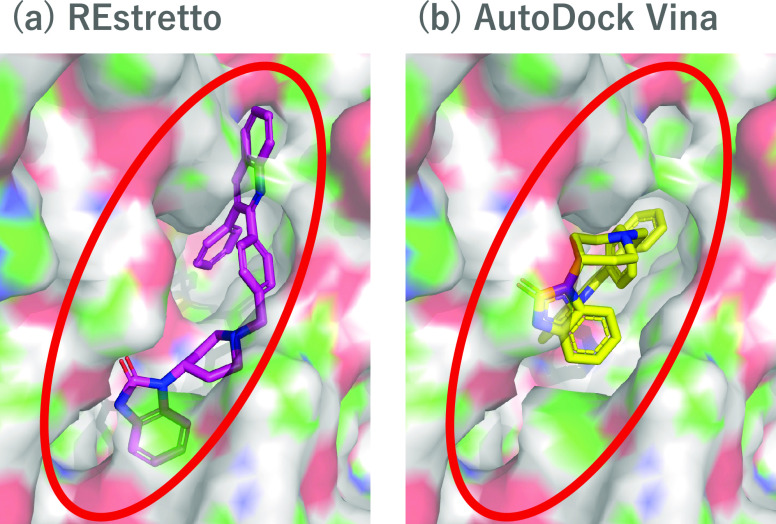
Docking pose examples docked by (a) REstretto and (b) AutoDock Vina. The compound is CHEMBL359864, which is known to be an active compound for the target protein AKT1.

As shown in Table S1, REstretto showed higher accuracy (ROC-AUC = 0.761) than AutoDock Vina (ROC-AUC = 0.566) for the AKT1 target. Figure 5 shows examples of docking poses obtained by using REstretto (Figure 5a, score: −11.6) and AutoDock Vina (Figure 5b, score: −3.9). The active compound was CHEMBL359864, for which REstretto scored the best among the compounds of the AKT1 target. Interestingly, the docking pose output by AutoDock Vina utilized only a part of the pocket, whereas that output by REstretto utilized the entire pocket. The cocrystallized ligand of the target was markedly smaller than the active compound (it only fits the deeper pocket of the binding site); thus, the search space determined by eBoxsize was also too small to cover the shallow region of the pocket. This fact considerably and negatively affected the results of AutoDock Vina because it does not place any atoms outside the search space. In contrast, the search space of REstretto was wider, as previously discussed, resulting in the achievement of a reasonable binding pose, even though the simplified fragment grids stored only the best scores among rotations. Additionally, the result also indicated that REstretto correctly placed the compound even if the best positions of fragments were distinct from each other. The search space estimated by Glide was also small, which explained the observed decreases in accuracy in both Glide HTVS (ROC-AUC = 0.533) and Glide SP (ROC-AUC = 0.564).

### Relationship between the Size of Compounds and the Accuracy of REstretto

In contrast to its success for AKT1, REstretto showed lower accuracy (ROC-AUC = 0.646) than AutoDock Vina (ROC-AUC = 0.749) in the human immunodeficiency virus type 1 protease (HIVPR) target, as shown in Table S1. The average number of atoms and average rotatable bonds per compound were calculated for each target of the DUD-E diverse subset (Table S4). The target HIVPR had the highest number of both the average number of heavy atoms (32.2–36.3) and the average rotatable bonds (8.5–9.6). The docking accuracy of REstretto may therefore be lower for relatively large number of heavy atoms and internal degrees of freedom of compounds. This may be due to the limited consideration of internal degrees of freedom and/or the number of sampling angles of rotation of REstretto. We employed conformer docking in our strategy; however, in the future, the prediction accuracy can be improved by a combination of a flexible ligand docking with incremental construction strategy and the prior knowledge of feasible compound conformations, which is used in conformer generators.

REstretto utilizes pregenerated conformers and does not change the conformation of the compounds; this aspect restricts the internal degrees of freedom of the compounds. Even though the maximum number of conformers was set to 200 in the present study, it might be difficult to generate the correct conformation of such large and flexible compounds; thus, the docking scores of compounds with many internal degrees of freedom tended not to be as high as the optimal values. Possible solutions to this problem include increasing the number of initial conformers depending on the internal degree of freedom of a compound and conducting local optimization with the flexibility of the compounds.

Another possible reason is the sampling granularity of the rotation angles. Although REstretto covers a wider physical search space (translations and rotations), the granularity of the search (interval of translation, number of sampling of rotation angles) is constant regardless of the score or the size of the compound. In particular, the displacement of atoms with constant rotation angles is highly dependent on the distance from the center of the compound selected. This may be resolved by employing a smaller step size of the rotation angle, especially for compounds with longer radii, which can cause a large displacement of atoms. However, the balance between accuracy and speed must be considered.

### Relationship between EF and the Local Optimization Method

We compared the performance of REstretto and AutoDock Vina in terms of two metrics, namely, ROC-AUC and EF. REstretto was superior to AutoDock Vina with respect to ROC-AUC, whereas it was inferior to AutoDock Vina with respect to EF. One possible reason for this is the differences in the local optimization step between each approach. REstretto optimizes translation and rotation from the initial docking poses, whereas AutoDock Vina optimizes the conformation (dihedral angles of a compound), translation, and rotation. The conformational space is large because a compound contains several rotatable bonds. Thus, AutoDock Vina may fail to identify near-optimal poses for many compounds, resulting in a relatively low ROC-AUC. Meanwhile, docking poses and conformations of active compounds are likely to be optimized to fit to a protein surface, resulting in higher EFs.

## Conclusion

In this study, we propose another virtual screening-oriented, fragment-based docking strategy with the following three factors for acceleration: compound decomposition, simplified fragment grid generation, and flexibility consideration with conformers. The compound decomposition is responsible for the generation of fragments that are common among many compounds. The use of the fragment grid of the intermediate result of a fragment substantially decreases the computational costs based on the fragment commonality. An issue with the fragment grid was memory consumption; however, the simplified fragment grid storing *k*-best scores solves the issue. Interestingly, the use of the simplified fragment grid (*k* = 1) hardly decreased the prediction accuracy while reducing the memory consumption to one-sixties. The flexibility consideration of a compound is the last challenge. Considering the flexibility using conformers enables us to dock a compound via a rigid docking procedure. This approach renders convenience to consistent reconstruction based on the feasible conformation space attached to the compound.

To assess the effectiveness of the procedure, we implemented REstretto, a proof-of-concept that uses the above-described fragment-based strategy employing the AutoDock Vina scoring function. We compared the performances of REstretto and AutoDock Vina in terms of their accuracy and speed using the DUD-E diverse subset. REstretto achieved comparable performance to that of AutoDock Vina. In addition, the calculation time of the fragment grid generation of REstretto is also reduced with an increase in the number of compounds because of the fragment commonality. These facts strongly support the effectiveness of the proposed strategy, especially in virtual screening for the evaluation of billions of compounds.

The more efficient implementation of the tool based on the proposed strategy is the next step of this research. For instance, REstretto assumed a single conformer per fragment even for macro rings having restricted but considerable flexibility. This issue regarding the prediction accuracy may be solved by generating a conformation-aware simplified fragment grid with the best scores among fragment conformations. Furthermore, a gradient-based local optimization enhances the calculation speed, which have already been observed with our preliminary implementation of it. These improvements will lead to the development of a more effective tool for structure-based virtual screening.

## Data and Software Availability

Compound structures and protein structures were obtained from the Directory of Useful Decoys, Enhanced (DUD-E). Implementation of REstretto is open-sourced under an MIT license at https://github.com/akiyamalab/restretto (accessed on August 4, 2022). We used OMEGA (version 2.5.1.4) to generate conformers and LigPrep (on Schrödinger suite, version 2018–1) to generate stereoisomeric and ionization states. The docking space of each target protein was determined using eBoxSize (version 1.1). Two docking tools, AutoDock Vina (version 1.1.2) and Glide (on Schrödinger suite, version 2018–3), were used to evaluate the effectiveness of the proposed procedure. Open-sourced PyMOL was used for the visualization.

## Supporting Information Available

Text S1, analysis of time complexity; Tables S1–S3, ROC-AUC and EF values for each target; Table S4, average number of atoms and the average number of rotatable bonds of each active/decoy set for each target; Table S5, ROC-AUC comparison between the use of full fragment grid and the use of simplified fragment grid; Figures S1–S8, ROC curves for five docking methods (REstretto, AutoDock Vina (ex = 1), AutoDock Vina (ex = 8), Glide SP, and Glide HTVS) for each target (PDF)

## Abbreviations

AKT1: serine/threonine-protein kinase AKT
AMPC: AmpC β-lactamase
AUC: area under the curve
CP3A4: cytochrome P450 3A4
CPU: central processing unit
CXCR4: C-X-C chemokine receptor type 4
EF: enrichment factor
GCR: glucocorticoid receptor
HIVPR: human immunodeficiency virus type 1 protease
HIVRT: human immunodeficiency virus type 1 reverse transcriptase
KIF11: Kinesin-like protein 1
PDB: Protein Data Bank
SBVS: structure-based virtual screening
RAM: random access memory
ROC: receiver operating characteristic
