# Supplementary material for: Effective Protein–Ligand Docking Strategy via Fragment Reuse and a Proof-of-Concept Implementation
Source: ACS Omega. 2022 Aug 19;7(34):30265–74. doi: 10.1021/acsomega.2c03470 (PMC9435046; doi:10.1021/acsomega.2c03470)
Supplement: Supplementary file 1 — ao2c03470_si_001.pdf [file ao2c03470_si_001.pdf]

Supporting Information: Effective protein–ligand docking strategy via fragment reuse and a proof-of-concept implementation

Keisuke Yanagisawa,[†,¶] Rikuto Kubota,[†,‡,¶] Yasushi Yoshikawa,[†] Masahito Ohue,[†] and Yutaka Akiyama[*,†]

†*Department of Computer Science, School of Computing, Tokyo Institute of Technology, Meguro-ku, Tokyo 152-8550, Japan*

‡*AIST-TokyoTech Real World Big-Data Computation Open Innovation Laboratory (RWBC-OIL), National Institute of Advanced Industrial Science and Technology, Tsukuba, Ibaraki 305-8560, Japan*

¶*Contributed equally to this work*

E-mail: akiyama@c.titech.ac.jp

Supporting Texts

Supporting Text S1: Analysis of Time Complexity

Here, we assumed a protein-ligand rigid docking. Following the use of the atom grids, the time complexities of the generation of atom grids and the evaluation of the compounds are $O\left(\sum_{a \in A} 1\right) = O\left(|A|\right)$, and $O\left(|T_A|\right)$, respectively, where A represents the types of atoms and $|T_A|$ represents the total number of atoms among all compounds. Since $|A| \ll |T_A|$ holds, the total time complexity of the atom grid-based docking is $O\left(|T_A|\right)$.

To estimate the time complexity of fragment reuse-based docking, two assumptions are introduced: (1) the time complexity of the generation of an intermediate result of a fragment f is proportional to the number of atoms in the fragment $O\left(|A_f|\right)$, and (2) the time complexity of the evaluation of a compound c is proportional to the number of fragments in the compound $O\left(|F_c|\right)$. Under these conditions, the time complexities of the generation of atom grids, the generation of the intermediate results of the fragments, and the evaluation of compounds are $O\left(|A|\right)$, $O\left(\sum_{f \in F} |A_f|\right)$, and $O\left(\sum_{c \in C} |F_c|\right) = O\left(|T_F|\right)$, respectively, where F represents the types of fragments presenting in compounds, C represents the set of compounds, $|A_f|$ represents the number of atoms in a fragment f, and $|T_F|$ represents the total number of fragments among all compounds. Since $|A| < O\left(\sum_{f \in F} |A_f|\right) \ll |T_F|$, the total time complexity of the fragment reuse-based docking is $O\left(|T_F|\right)$. It is noteworthy that the use of the fragment grid as the intermediate result of a fragment fulfills the two assumptions.

Supporting Tables

Table S1: ROC-AUC metrics for each target in the DUD-E diverse subset. The best values between REstretto and AutoDock Vina are shown in bold.

Target	REstretto	AutoDock Vina (ex = 1)	AutoDock Vina (ex = 8)	Glide HTVS	Glide SP
	ROC-AUC				
AKT1	**0.765**	0.566	0.604	0.533	0.564
AMPC	**0.590**	0.571	0.566	0.790	0.832
CP3A4	**0.603**	0.589	0.587	0.654	0.672
CXCR4	**0.632**	0.577	0.573	0.729	0.666
GCR	**0.605**	0.554	0.575	0.509	0.617
HIVPR	0.646	**0.749**	0.742	0.683	0.818
HIVRT	0.638	**0.666**	**0.666**	0.638	0.769
KIF11	0.779	0.832	**0.841**	0.801	0.861
Average	**0.657**	0.638	0.644	0.667	0.725

Table S2: The $EF_{1\%}$ metrics for each target in the DUD-E diverse subset. The best values between REstretto and AutoDock Vina are shown in bold.

Target	REstretto	AutoDock Vina (ex = 1)	AutoDock Vina (ex = 8)	Glide HTVS	Glide SP
	$EF_{1\%}$				
AKT1	**3.8**	0.3	1.4	4.1	10.6
AMPC	**2.1**	0.0	0.0	2.1	6.3
CP3A4	**2.4**	2.4	1.8	5.3	8.8
CXCR4	0.0	0.0	0.0	0.0	5.0
GCR	12.4	12.8	**15.1**	12.0	16.7
HIVPR	2.1	4.3	**5.2**	9.3	20.1
HIVRT	5.0	**7.4**	**7.4**	13.6	22.2
KIF11	14.7	30.2	**31.9**	22.4	42.2
Average	5.3	7.2	**7.8**	8.6	16.5

Table S3: The $EF_{10\%}$ metrics for each target in the DUD-E diverse subset. The best values between REstretto and AutoDock Vina are shown in bold.

Target	$EF_{10\%}$ REstretto	AutoDock Vina (ex = 1)	AutoDock Vina (ex = 8)	Glide HTVS	Glide SP
AKT1	**3.3**	1.9	2.1	1.2	2.3
AMPC	0.8	1.3	**1.5**	3.7	5.6
CP3A4	**1.9**	**1.9**	1.7	2.5	3.3
CXCR4	**1.3**	0.8	0.8	3.3	1.5
GCR	2.8	3.0	**3.3**	2.1	3.6
HIVPR	2.8	**3.4**	3.2	3.8	5.7
HIVRT	2.7	**2.9**	2.7	3.2	5.2
KIF11	5.5	6.1	**6.4**	5.3	7.0
Average	2.6	**2.7**	**2.7**	3.1	4.3

Table S4: Average number of atoms and the average number of rotatable bonds of each active/decoy set for each target. The greatest numbers are shown in bold.

Target		Average number of atoms	Average number of rotatable bonds
AKT1	Actives	33.5	6.6
	Decoys	28.9	7.3
AMPC	Actives	19.9	4.0
	Decoys	20.4	4.1
CP3A4	Actives	31.7	7.1
	Decoys	29.4	6.9
CXCR4	Actives	28.4	6.4
	Decoys	25.0	6.6
GCR	Actives	32.1	4.3
	Decoys	27.6	5.2
HIVPR	Actives	**36.3**	**9.6**
	Decoys	**32.2**	**8.5**
HIVRT	Actives	25.8	4.9
	Decoys	24.2	4.9
KIF11	Actives	29.7	6.2
	Decoys	26.1	5.5
All targets	Actives	32.3	7.0
	Decoys	28.5	6.7

Table S5: ROC-AUC values obtained with the use of full fragment grids and those obtained with the use of simplified fragment grids for five targets in the DUD-E

Target	full fragment grid	simplified fragment grid
LKHA4	0.774	0.790
PNPH	0.859	0.850
PUR2	0.549	0.547
PYGM	0.531	0.522
WEE1	0.908	0.929

Supporting Figures

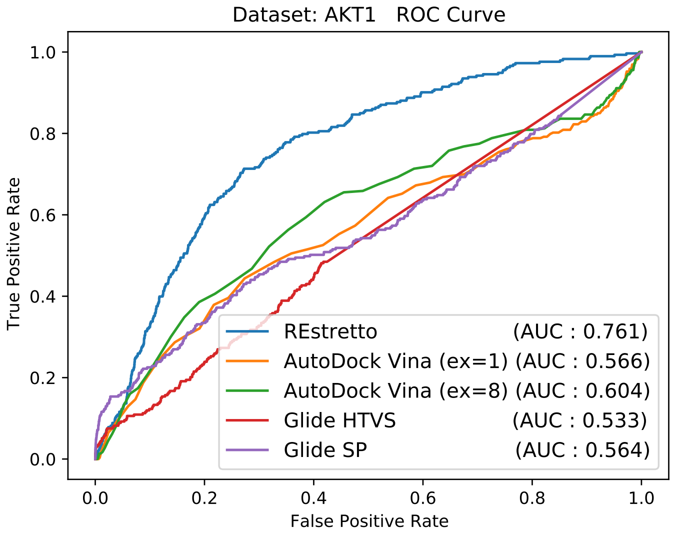

Figure S1: ROC curves for five docking methods for the target AKT1.

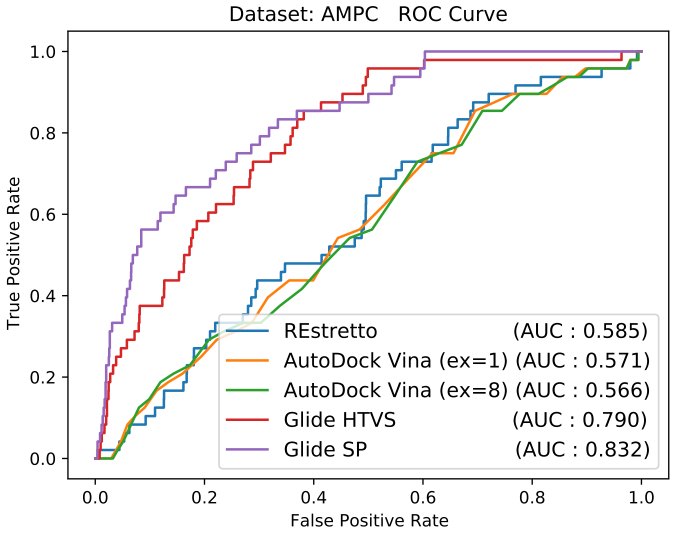

Figure S2: ROC curves for five docking methods for the target AMPC.

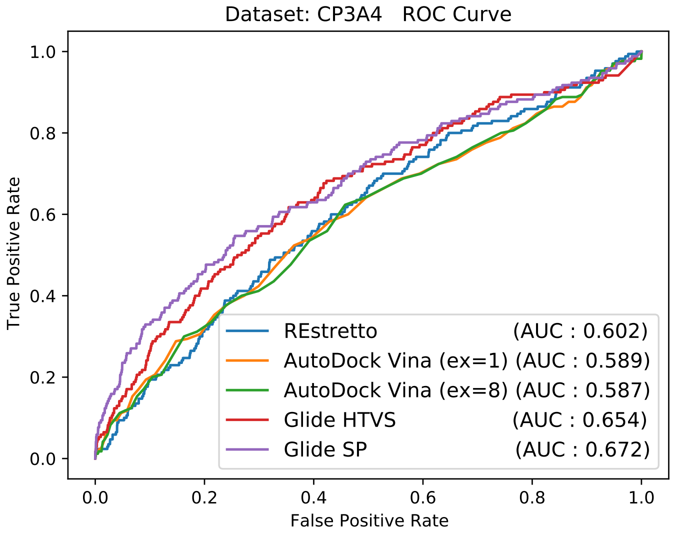

Figure S3: ROC curves for five docking methods for the target CP3A4.

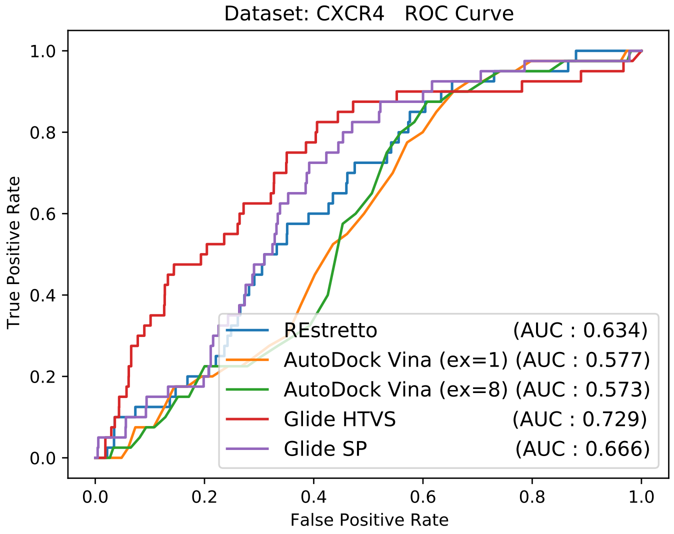

Figure S4: ROC curves for five docking methods for the target CXCR4.

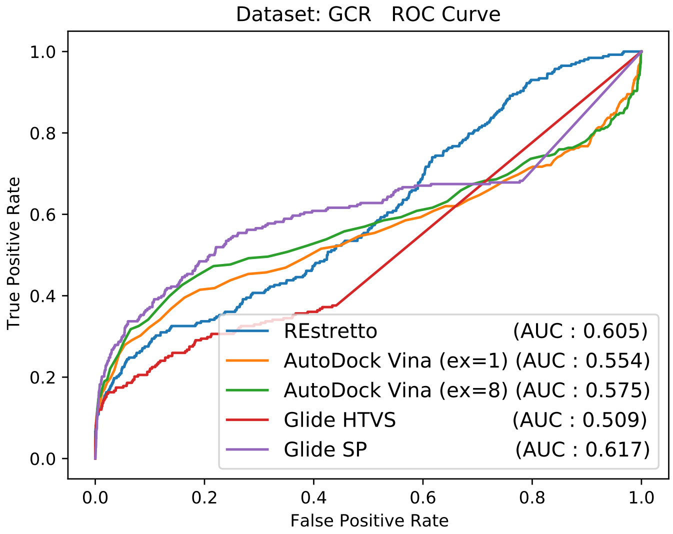

Figure S5: ROC curves for five docking methods for the target GCR.

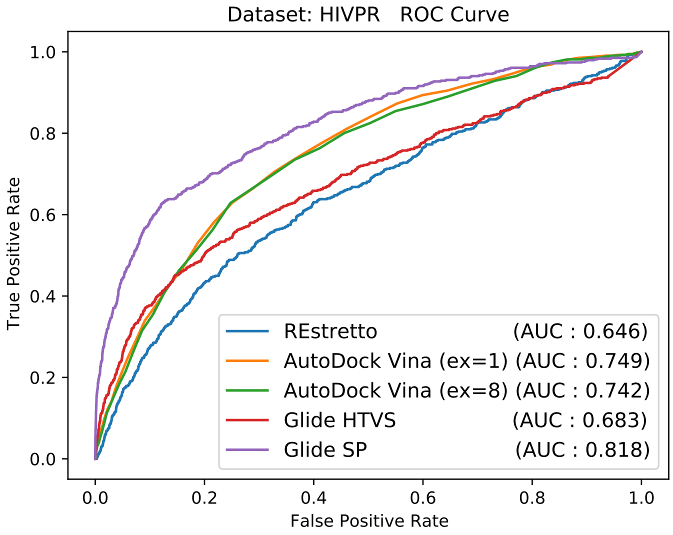

Figure S6: ROC curves for five docking methods for the target HIVPR.

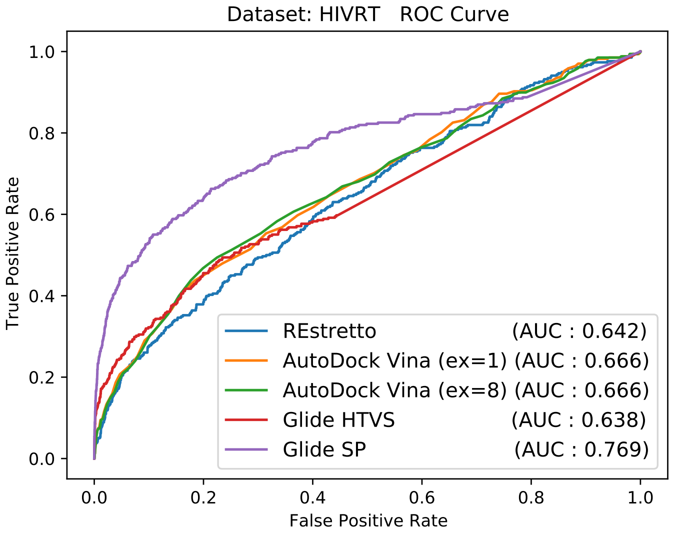

Figure S7: ROC curves for five docking methods for the target HIVRT.

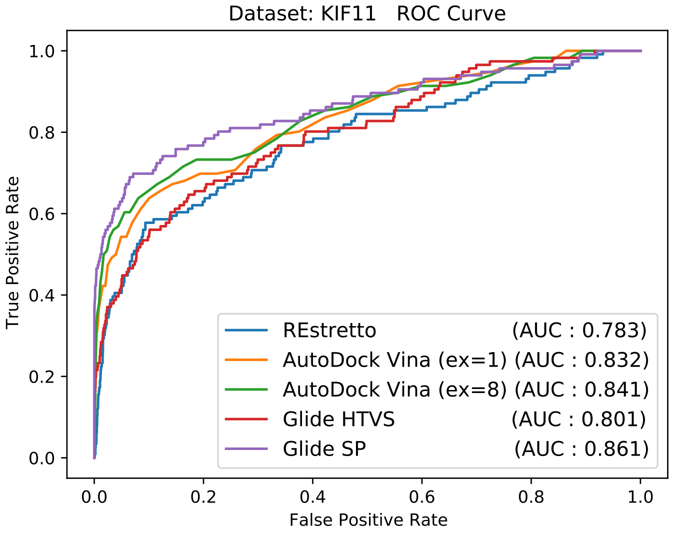

Figure S8: ROC curves for five docking methods for the target KIF11.
